# Nanostructured Chitosan-Based Biomaterials for Sustained and Colon-Specific Resveratrol Release

**DOI:** 10.3390/ijms20020398

**Published:** 2019-01-18

**Authors:** Nieves Iglesias, Elsa Galbis, M. Jesús Díaz-Blanco, Ricardo Lucas, Elena Benito, M.-Violante de-Paz

**Affiliations:** 1 Dpto. Química Orgánica y Farmacéutica, Facultad de Farmacia, Universidad de Sevilla, 41012-Seville, Spain; nievesiglesias@us.es (N.I.); elsa@us.es (E.G.); rlucas1@us.es (R.L.); ebenito@us.es (E.B.); 2 PRO2TECS. Departamento de Ingeniería Química, Facultad de Ciencias Experimentales, Campus El Carmen–21071-Huelva, Spain; dblanco@diq.uhu.es

**Keywords:** colon, inflammatory bowel disease, IBD, drug depot, mucoadhesive, Crohn’s disease, ulcerative colitis, nanoparticles, hydrogels

## Abstract

In the present work, we demonstrate the preparation of chitosan-based composites as vehicles of the natural occurring multi-drug resveratrol (RES). Such systems are endowed with potential therapeutic effects on inflammatory bowel diseases (IBD), such as Crohn’s disease (CD) and ulcerative colitis, through the sustained colonic release of RES from long-lasting mucoadhesive drug depots. The loading of RES into nanoparticles (NPs) was optimized regarding two independent variables: RES/polymer ratio, and temperature. Twenty experiments were carried out and a Box–Behnken experimental design was used to evaluate the significance of these independent variables related to encapsulation efficiency (EE). The enhanced RES EE values were achieved in 24 h at 39 °C and at RES/polymer ratio of 0.75:1 *w*/*w*. Sizes and polydispersities of the optimized NPs were studied by dynamic light scattering (DLS). Chitosan (CTS) dispersions containing the RES-loaded NPs were ionically gelled with tricarballylic acid to yield CTS-NPs composites. Macro- and microscopic features (morphology and porosity studied by SEM and spreadability), thermal stability (studied by TGA), and release kinetics of the RES-loaded CTS-NPs were investigated. Release patterns in simulated colon conditions for 48 h displayed significant differences between the NPs (final cumulative drug release: 79–81%), and the CTS-NPs composites (29–34%).

## 1. Introduction

Although inflammation is part of the normal host response to infection and injury, excessive or inappropriate inflammation contributes to a range of acute and chronic human diseases which are characterized by the production of inflammatory cytokines, arachidonic acid–derived eicosanoids, other inflammatory agents and adhesion molecules [1]. Thus, high concentrations of Tumor necrosis factor (TNF-α), Interleukin-12 (IL-12), and Interleukin-6 (IL-6) are particularly destructive and are implicated in some of the pathologic responses that occur in endotoxic shock, in acute respiratory distress syndrome, and in chronic inflammatory diseases such as rheumatoid arthritis and inflammatory bowel disease (IBD) [2].

IBD is a group of disorders characterized by a chronic and relapsing inflammation of the gastrointestinal tract frequent in Western countries [3]. The two most common forms of IBD are Crohn’s disease (CD) and ulcerative colitis (UC). They lead to long-term and sometimes irreversible impairment of gastro-intestinal structure and function [4]. In UC, a diffuse mucosal inflammation of the colon is mainly found as well as the coincident production of a complex mixture of inflammatory mediators and extensive superficial mucosal ulceration. Conversely, in CD, any part of the gastro-intestinal tract (GIT) from the mouth to the anus can be affected, although it is usually the ileum and colon. Unlike UC, CD may be patchy and segmental. In addition, inflammation can be typically transmural [1,2].

It has been hypothesized that IBD results from an inappropriate and exaggerated mucosal immune response to normal constituents of the mucosal microflora that is, in part, genetically determined [2]. It is now well established that microbial components of the resident microbiota can regulate gut inflammation [5]. In CD, in particular, the intestinal microbiota is strongly suspected to play a role in initiating and triggering the immune system, leading to characteristic inflammation [6,7]. However, the inheritable component also plays a role and seems stronger in CD than in ulcerative colitis [8]. Moreover, it is worth highlighting that in several countries with historically low rates of IBD, a pattern of rising incidence in the past one to two decades, particularly for CD, has occurred, suggesting that environmental factors are also involved [4].

**Figure 1:**
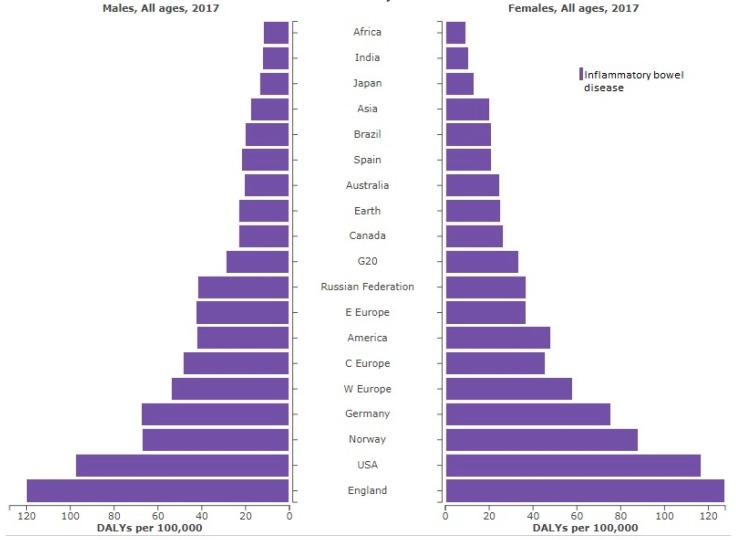
DALYs (disability-adjusted life years) per year of inflammatory bowel disease (IBD) in selected locations in 2017, ordered by incidence and sex.

IBD has an enormous impact on people’s lives. According to the Institute for Health Metrics and Evaluation (GBD 2017, University of Washington) IBD was responsible, globally, for the 0.07% of deaths that occurred in the world in 2017, i.e., the death of 3.86 million people was directly correlated with some of these pathologies. However, they also have a marked influence on the quality of life of ill people. Thus, the global disability-adjusted life years (DALYs) connected with these disorders was of 184.95 million in 2017. The highest DALY values were found for people from England, the United States of America and Norway, as can be seen in Figure 1. Therefore, finding new therapies aimed at the overall reduction of the incidence of IBD is a necessity.

Conventional treatment of IBD is based on the daily administration of high doses of immune-suppressant or anti-inflammatory drugs, often complicated by serious adverse effects. Thus, a carrier system that delivers the drug specifically to the inflamed intestinal regions and shows prolonged drug release would be desirable [9].

Resveratrol (RES), a natural occurring polyphenol, is the main biologically active component in red wine. Hundreds of publications have demonstrated that RES can prevent or delay the progression of a wide variety of diseases [10] due to its antiplatelet activity [11] as well as antitumor [12], neuroprotective [13,14] and anti-inflammatory [15,16] properties. Resveratrol has been reported to decrease inflammation by inhibiting the induced production of pro-inflammatory cytokines, such as TNF-α, IL-1β, IL-6, and IL-8, and matrix metalloproteinases MMP-2, MMP-3, MMP-9 and MMP-13, in in vitro and in vivo models [17]. The main problem associated with the therapeutic use of RES is its rapid metabolism in vivo [18] and it is related to its low water solubility and chemical instability. This drawback has been addressed by the preparation of resveratrol derivatives so that their intrinsic biological activity and bioavailability would be improved [17,19,20]. 

The development of colon-targeting drug delivery systems (DDS) is one of the most grown fields in pharmaceutical technology. They can be employed for both local-acting drugs and active principles intended for systemic absorption. These colon specific drug delivery systems are gaining importance for use in the treatment of colon cancer, chronic disorders, such as IBD and also for the systemic delivery of protein and peptide drugs. Ferris et al. reported the preparation of reduction-sensitive biodegradable multiallyl- and multiamine-based copolyurethanes for their use as colon-targeted anticancer drug carriers [21]. Xu et al. [22] prepared dual crosslinked gel beads composed of alginate and chitosan for the colonic site-specific delivery of bovine serum albumin [23]. Peppas and colleagues have reported the preparation of responsive hydrogels for the treatment of the irritable bowel syndrome [24]. Hydrogel patches that contain nanoparticles (NPs) loaded with siRNA and a chemo drug were capable of preventing the recurrence of colon cancer in a murine model [25]. Dextran-based hydrogels have been investigated as colon-targeted drug delivery systems due to the presence of dextranase in the colon [26]. Resveratrol formulations of Zn-pectin-chitosan microparticles have demonstrated colon-specific drug release [27]. The use of micro-nanoparticles (MNP) in therapy of IBD with enhanced colonic retention of drugs has been reported [28].

In the current paper and based on our previous experience, we aim at the preparation of nanostructured chitosan-based hydrogels as a vehicle for RES in order to improve its ADME-T properties (absorption, distribution, metabolism, excretion and toxicity) for its final use in IBD. RES will be encapsulated into recently designed biocompatible non- and cross-linked poly(2-hydroxyethyl methacrylate)-based NPs as controlled DDS [29]. They have already demonstrated its capability of solubilizing lipophilic molecules such as pyrene, pilocarpine and the anticancer drug camptothecin [29,30,31,32]. The optimization conditions for loading RES into the NPs will be determined. The RES-loaded NPs will then be embedded into a shear-thinning chitosan-based hydrogel to form micro- and nano-structured smart hydrogel composites with bioadhesion properties as a drug depot. The release studies of RES will be conducted in simulated colonic fluids from both the NP systems and the hydrogel nanocomposites.

## 2. Results and Discussion

In general terms, polymeric DDSs enhance the solubility of scarcely soluble drugs, improving their biodistribution and pharmacokinetics, and minimizing the side effects of the therapeutic molecules under study [33]. In the current work, the therapeutic molecule RES has been loaded into amphiphilic NPs that have demonstrated a highly efficient encapsulation efficiency for other lipophilic drugs such as pyrene, pilocarpine and camptothecin [29,30,31,32]. The hydrophilicity of the shells ensures the stability of the NPs in aqueous media. In the present work, the hydrophilic layer of the NPs is constituted by the biocompatible and widely used poly(2-hydroxyethyl methacrylate) (pHEMA) and the pH sensitive poly(*N,N*-dimethylaminoethyl methacrylate) (pDMAEMA). pHEMA is a non-toxic and biocompatible hydrophilic material and it is particularly attractive for biomedical engineering applications [34] as well as pDMAEMA, polymer used in the co-delivery of paclitaxel and DNA [35] with pH responsive behavior [36]. 

It was been stated that the instability associated to the micelle-unimers equilibrium by dilution in human fluids could lead to premature drug release in off-targeted tissues [37]. In order to study the influence of dilution on the prepared RES-loaded NPs, a double study has been conducted with non-stabilized NPs and with a batch of cross-linked, hence, stabilized NPs [29]. 

### 2.1. Resveratrol-Loaded Nanoparticules

To optimize the loading of RES into the freshly prepared NPs, the variables investigated were those that displayed the most marked effect on the encapsulation efficiency (EE) of the drug camptothecin by these systems [32], i.e., drug/polymer ratio, and temperature. 

The notable influence of drug/polymer ratios in the feed on the EE of hydrophobic molecules into polymer NPs has been confirmed in multiple studies. In general terms, it has been observed that the bigger the drug concentration in the feed, the higher was its EE [34,38,39,40,41]. For the experimental design, RES-polymer ratios were set to values of 0.25, 0.50 and 0.75 (*w*/*w*). The range of temperature chosen for the current experimental design (from 25 °C to 39 °C) was selected based on the most common laboratory working temperature (25 °C) and with the objective of keeping the hydrophilicity of the shells, i.e., avoiding them reaching the lower critical solution temperature (LCST) of pDMAEMA (42 °C [42]), the main component of NP shells. 

Table 1 shows the values of the independent variables and the experimental values of the EE obtained for both the non- and the cross-linked NPs. 

In Table 2, the obtained equations calculated using polynomial regression and statistics parameters are shown. 

The obtained equations show acceptable (> 0.96) R^2^, (> 25) F and (> 0.005) *p*-values, the reason why a suitable modelling is proved. However, in both equations, complex terms involving interactions between independent variables have been calculated.

Moreover, identifying the independent variables most and least strongly influencing the dependent variables in equations showed in Table 2 is not straightforward since those equations contain interactions between two independent variables terms. A Pareto chart, also called a Pareto distribution diagram, is used for analyzing what independent variables (*p* > 0.05) have the greatest cumulative effect on this study. Figure 1 shows a plot of each dependent variable (compound) and its Pareto chart of standardized effects (as percentages) based on independent variables. 

**Figure 2:**
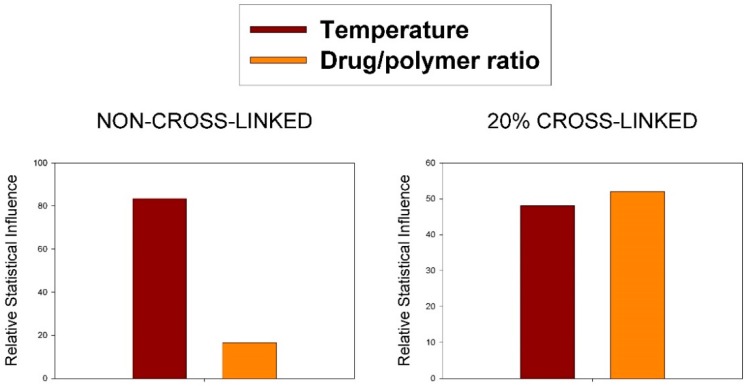
Relative influence of the studied variables on encapsulation efficiency (EE%) in non-cross-linked and cross-linked nanoparticles.

**Figure 3:**
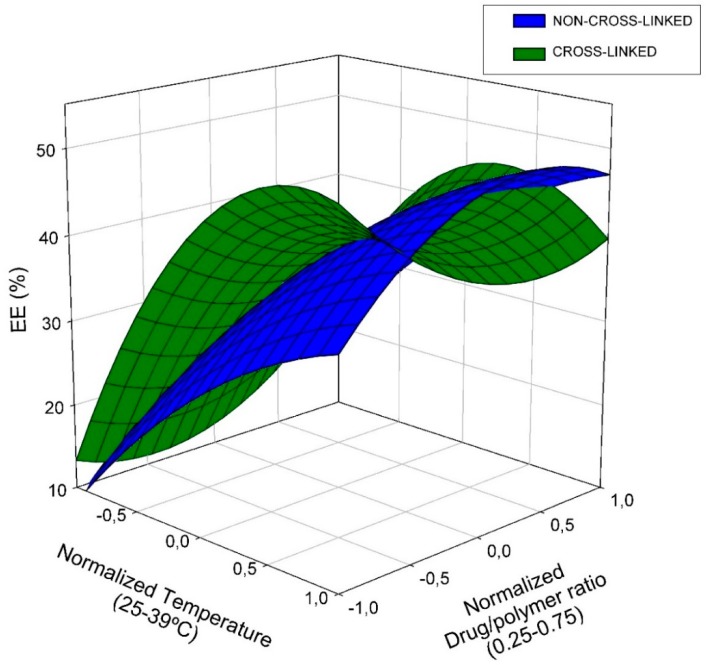
Response surface for RES encapsulation efficiency percentage on both, non- and cross-linked NPs.

The Pareto charts (Figure 2) show that the most important variable on non-cross-linked EE evolution is temperature (83%); it happens, likewise, on camptothecin loaded NPs [32]. However, similar relative statistical influence (48% and 52% for temperature and drug/polymer ratio respectively) has been found for 20% cross-linked treatment. In general terms, as observed previously for other systems, the higher the drug/polymer ratio, the greater the EE. The EE values are also in agreement with those obtained for camptothecin at similar drug/polymer ratios. To help the interpretation of the obtained equations, the response surface for each dependent variable is shown (Figure 3). 

The RES-loaded NPs with optimized EE (Table 3) were studied by DLS. The Z-average and D_h_ of the non-cross-linked system experienced a slight reduction compared to the unloaded counterpart. This could be due to the π–π tacking of the drug molecules and the furfuryl rings of the core of the NP, causing more compact micelles. In the case of cross-linked NP loaded with RES, the size became slightly higher than the unloaded corresponding NP, as has been observed for other systems [31,32]. 

### 2.2. Nanostructured Chitosan-Based Composites Containing Resveratrol-Loaded NPs

Hydrogels are currently being studied as matrices for the controlled release of bioactive molecules, and for the encapsulation of living cells. For these applications, it is often required that the hydrogels degrade under physiological conditions, i.e., the originally three-dimensional structures have to disintegrate preferably in harmless products to ensure a good biocompatibility of the biomaterial. 

Recently, much attention has been paid to chitosan (CTS) because of its advantageous biological properties such as biodegradability, biocompatibility and non-toxicity as well as its physicochemical properties [43]. Thus, CTS and its derivatives have been used as an absorption enhancer [44], drug carrier [45,46], mucoadhesive and permeation enhancing polymer in formulations for buccal/sublingual, nasal, gastrointestinal, vaginal, colonic drug delivery [47] and for gene delivery [48]. 

Chitosan has been found to be degradable by the colon microflora. Taking advantage of this property, CTS has turned out to be a useful material to guarantee the colonic drug delivery as long as it is part of the controlled drug release systems [49]. These materials can adhere tightly to mucosal surfaces such as gastrointestinal tract (GIT) walls and transiently opening the tight junction between epithelial cells, enhancing drug absorption across intestinal epithelial cells without injuring them [50]. Thus, for example, sustained intestinal delivery of drugs such as 5-fluorouracil and insulin seems to be a feasible alternative to injection therapy [51]. In addition, the bioadhesiveness property of chitosan-based DDS has made its formulations useful as local drug-depots [52]. The above-mentioned properties make CTS an ideal polymer for colon-specific drug release and it was the biocompatible polymer chosen in the present work. 

In general terms, polymeric network formation can be accomplished either by non-covalent physical associations, such as secondary forces (hydrogen, ionic, or hydrophobic bonds) and physical entanglements, or by covalent cross-links. To form stabilizing linkages in chitosan-based materials, the amine moieties present in their structure allow the linkage between the chains through selected cross-linkers to prevent gel dissociation. 

In a previous work, we have carried out the synthesis of micro-structured biomaterials based on ionically cross-linked CTS for their applications as biocompatible carriers of drugs and bioactive compounds. The influence of the dispersion composition on its rheological properties was evaluated. The release profiles of a model drug, diclofenac sodium (DCNa) as well as their relationships with polymer concentration, drug loading and degree of cross-linking were established [53]. Based on this study, we have carried out the preparation of CTS-based bioadhesive hydrogels with RES-loaded NP embedded into their structure. In this case, part of the distilled water necessary for the hydrogel formation has been replaced by an optimized RES-loaded NP suspension. 

The spreadability of the systems as well as their morphologies and thermogravimetric analyses of the freeze-dried samples were investigated. Surprisingly, the macroscopic properties of the composites were similar to those without the inclusion of the nanoparticles. Table 4 displays some relevant data from the systems studied. 

The mechanical properties of formulations designed for GIT administration are fundamental in composite performance. The spreadability of the product is one of them and contributes to the final clinical efficacy of the product [54]. On the other hand, the composites need the displaying mucoadhesion properties to act as an RES depot in the GIT. Mucoadhesion is controlled by the affinity of the material for the mucin glycoproteins of the mucus and CTS has demonstrated the exhibition of excellent mucoadhesive properties due to its amine and hydroxyl groups, which are involved in its prolonged residence time in the gastrointestinal tract [49]. The composite formulated in the present work displayed excellent spreadability properties as expected, which ensure the satisfactory application on the damaged tissue. From data obtained in the current and previous works, it can be concluded that the higher the CTS concentration in the formulations, the more structured hydrogels are obtained, and the lower the spreadibility found.

**Figure 4:**
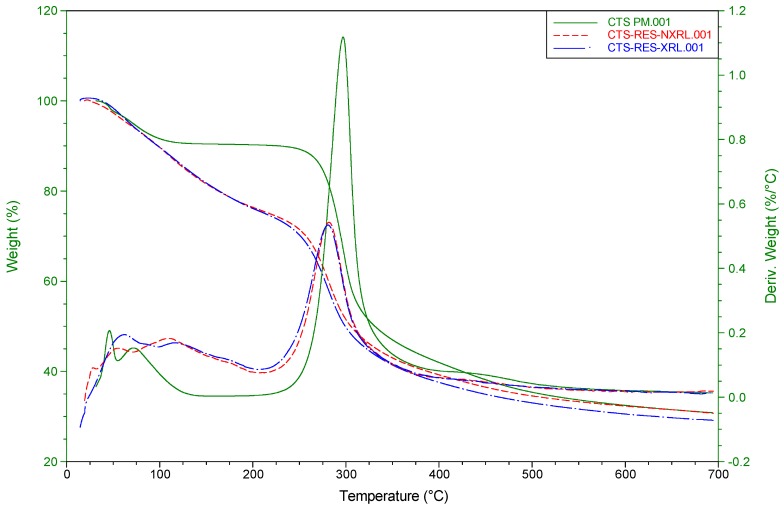
TGA traces of commercial chitosan (solid line) and the two RES-loaded CTS-NP composites.

A thermogravimetric study of the commercial CTS and the two freeze-dried CTS-NP conjugates was conducted in order to reveal the variations in thermal degradability between the starting material and the composites. Their traces are superimposed in Figure 4. For illustrative purposes, the curves that represent the derivative weight loss vs. temperature are also included.

The new chitosan-based conjugates display almost identical thermogravimetric profiles and show a maximum degradation temperature at 281 °C. Although this value is slightly shifted to lower temperatures compared with the starting material (297 °C), the peak profile is similar to CTS’s one, as can clearly be seen in their derivative thermogravimetric analysis (DTG) curves. The new formulations experience a reduction in mass of 24% at low temperatures in contrast with the commercial CTS (9%), which could be mostly due to water content in the CTS sample. 

**Figure 5:**
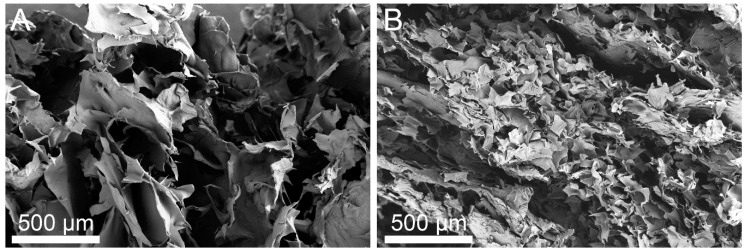
SEM images of the loaded chitosan-based hydrogels: (**A**) Xr and (**B**) Non-Xr.

As SEM has proved to be a particularly relevant technique to determine the scaffold characteristics (pore size and morphology) of hydrogels and biological systems, some SEM images were obtained in order to study the microstructure of the composites formed. To preserve the skeleton structure of our systems prior to SEM observations, the freeze dried method previously reported for cells has been used [53,55,56]. Figure 5 compares SEM micrographs of the chitosan-based hydrogels with RES-loaded Xr and non-Xr NPs. The two images taken at the same magnification show that both samples have a porous microstructure. It should be noted that, unlike the macroscopic properties, the microstructure of the two prepared conjugated differ in their 3D-micro-structure and the scaffold of the Xr sample displays larger pores.

### 2.3. Resveratrol Release Studies

The last part of the present work focused on evaluating the release of RES from the NPs and the CTS-NP composites in simulated colonic environments. Due to the highly-structured hydrogel scaffolds, the mechanisms of drug release from them are markedly different from those of other DDSs such as micelles and dendrimers. Previous modeling studies predicted that the release of an active agent from a hydrogel is determined by the rate-limiting step of the process and, therefore, categorized as diffusion-controlled, swelling-controlled, or chemically-controlled, being the former the primary mechanism that governs the release of drugs from hydrogels [49].

**Figure 6:**
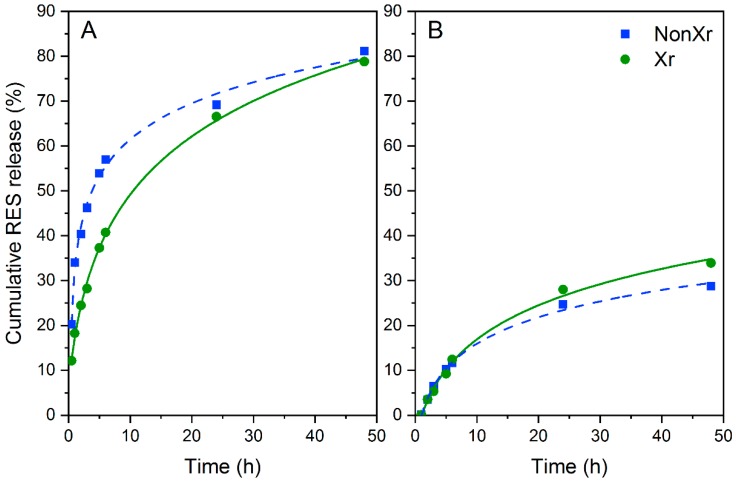
In vitro release profiles of resveratrol in simulated colon conditions from resveratrol-loaded NPs (**A**) and resveratrol-loaded CTS-NPs composites (**B**).

Both the NPs and the CTS-NPs composites were immersed in a simulated colonic environment at 37 °C and the drug release was evaluated by UV-Vis measurements at 307 nm. The cumulative RES release profiles of NPs and CTS-NPs composites are shown in Figure 6A,B, respectively.

The percentages of drug release were determined using Equation (1):

(1) $$Cumulative\;RES\;release\;(\%)\;=\frac{m_{entrapped\left(0\right)}-\;m_{residual\left(t\right)}}{m_{entrapped\left(0\right)}}\times 100$$

where $m_{entrapped\;\left(0\right)}$ is the weight of initial entrapped RES into the NPs; $m_{residual\;\left(t\right)}$ is the weight of residual RES at time “t” into the nano-microcarriers. 

For these trials, the use of normalized data was chosen in order to compare the capacity of the systems studied to retain or not the drug into the NPs (non- or cross-linked) on their own (Figure 6A) or immersed into a CTS network (Figure 6B).

The most significant fact observed was the similar behavior of both NPs studied, with a positive sustained release of RES over time. When the CTS-NPs composites were studied, it was be taken into account that cross-linkages in hydrogels could sufficiently restrain the hydrogel 3D-networks and the water flow within the systems. On the other hand, physical associations would rise to reversible bonds, labile over time and with definitive effects on the drug release kinetics of hydrogel-based DDS.

As expected, once the RES reservoir was immersed into a CTS network, a substantial decrement in RES release rates was observed, and hence most of the drug remained in the composite (final cumulative release (%) after 48 h: 29–34%), highlighting the marked influence of network environments on the drug release rates. These facts support the hypothesis that there are significant benefits associated with the design of drug depot systems based on nano-microstructured CTS biomaterials.

## 3. Materials and Methods

### 3.1. Materials

Resveratrol was provided by Segura’s Center of Edaphology and Applied Biology (CEBAS-CSIC) (Murcia, Spain). For the present work, a CTS sample (deacetylation degree = 75%) from Sigma-Aldrich (Saint Louis, MO, USA) has been chosen. CTS molecular weight (*M_v_*) was determined by viscometric analysis using an Anton Paar AMVn automated microviscometer. By means of the Mark–Houwink equation ([η] = 3.385 dL/g), *M_v_* was found to be 299 kDa [57]. The other chemicals used in the current work were purchased from Sigma-Aldrich and used as received. The 1 kDa cut-off mini-dialysis tubes used in the present work were purchased from GE Healthcare (Wauwatosa, WI, USA).

### 3.2. General Methods

Ultraviolet-visible (UV-Vis) measurements were carried out using a Shimadzu UV-2102 PC UV–visible spectrophotometer (Kyoto, Japan). The data were the result of, at least, three measurements.

The morphologies of the hydrogels were observed by field emission scanning electron microscopy. Before SEM observations, the samples scaffold were directly frozen at –20 °C for 3 h, then at –80 °C for 24 h. They were then lyophilized by freeze drying for 24 h. Finally, the dry hydrogel was fixed on aluminum stubs, coated with a thickness of about 10 nm of platinum-iridium (Pt-Ir), and imaged by scanning electron microscopy using a field emission FEI TENEO microscope (Hillsboro, OR, USA) operating at 5 kV at the General Research Services of the University of Seville (CITIUS). 

By using of a Malvern Zetasizer Nano ZS (Malvern Instruments, Malvern, UK) at 25 °C, the morphological parameters of the nanoparticles, i.e., the size distribution (polydispersity index, PdI) and the average diameter (Dh) were estimated by dynamic light scattering (DLS). Data for each sample were the result of the average from at least three measurements performed with a scattering angle of 173 ° to the incident beam, and figures were analyzed by means of a CONTIN algorithm.

The chosen samples were examined by thermogravimetric analysis (TGA), and the decomposition temperatures of the different samples could be observed. Thermogravimetric analyzer was TA Instruments Q-600 SDT (New Castle, DE, USA). Platinum pans containing approximately 5 mg of each sample were used. Trials were conducted under inert atmosphere (nitrogen, heating rate = 10 °C/min), from 0 °C to 700 °C. The spreadability of the nanostructured hydrogels was attained by measuring the extension area (diameter in cm, after 1 min and 30 min) of the sample when it is situated between two glass plates (20 cm × 20 cm) and exposed to a constant weight.

### 3.3. Micelle Formation

The nanoparticles were prepared from a synthesized *block*-copolymer with the molar composition poly[(DMAEMA_31%_-HEMA_19%_)-*block*-(DEAEMA_45%_-FMA_5%_)] (*M_n_* = 34,700; *M_w_* = 45,100; *M_w_/M_n_* = 1.3) [58] and some of the nanoparticles were cross-linked by Diels–Alder reaction, using 1,8-dimaleimide-3,6-dioxaoctane (DMDOO) as a cross-linker [59].

### 3.4. Preparation of Resveratrol-Loaded NPs

The general loading procedure was conducted as follows: the selected micellar dispersion was introduced into a mini-dialysis tube (1 kDa cut-off, GE Healthcare). The latter was placed into a sealed tube containing a freshly prepared aqueous-based RES solution (1:4 *v*/*v* DMSO-water) at a predetermined RES/polymer ratio and gently stirred for 24 h at 25, 32 or 39 °C. To determine the encapsulation efficiency in the loading processes, the remaining RES concentrations were measured at predetermined times by UV spectroscopy at 307 nm. 

### 3.5. Experimental Design to Study the Effect of Loading Conditions on Resveratrol Encapsulation 

The encapsulation studies of resveratrol by the freshly prepared non- and core cross-linked NPs were conducted by means of UV spectroscopy varying several experimental parameters such as RES/polymer ratio and temperature. The RES-loaded NPs with the optimized EE were also studied by DLS. These systems were the RES-loaded NP of choice to be embedded into CTS-based hydrogels. 

In order to obtain optimized conditions for the loading step, a Box–Behnken experimental design (CSS Statistica, StatSoft Inc., Tulsa, OK, USA) was used to evaluate the significance of the independent variables (temperature and resveratrol/polymer ratio), as well as the interactions among them in the non- and core cross-linked NPs. This experimental design [60,61] enabled the construction of second-order polynomials for each independent variable and the identification of statistical significance in the variables. For two variables, 10 experimental points are established.

Independent variables were normalized by using Equation (2):

(2) $$X_{n}=\;\frac{X\;-X_{med}\;}{\left(X_{max}-\;X_{min}\right)\;/\;2}$$

where $X_{n}$ is the normalized value of independent variables; X is the absolute experimental value of the variable concerned; $X_{med}$ is the mean of all fixed values for the variable in question; and $X_{max}$ and $X_{min}$ are the maximum and minimum values of the variable, respectively.

To study the influence of RES/polymer ratio and temperature in the systems, 20 RES-loaded NP systems, 10 of them named NonXr-RES/POL_x_-T_y_ and the other 10 systems named Xr-RES/POL_x_-T_y_ were prepared from non cross-linked NPs and cross-linked NPs, respectively. The final polymer concentration was 0.1 mg/mL, the final targeted RES/polymer ratios were 0.25, 0.50 and 0.75, and the loading temperatures were 25 °C, 32 °C and 39 °C. 

Encapsulation efficiency of RES embedded into the nanoparticles were calculated according to Equation (3):

(3) $$EE=\;\frac{mass\;of\;RES\;loaded\;into\;NP}{mass\;of\;RES\;at\;t_{0\;}in\;the\;incubation\;tube}\times 100$$

### 3.6. Preparation of Nanostructured Chitosan-Based Hydrogels with Resveratrol-Loaded Nanoparticles

Two systems were prepared with CTS of molecular weight 199 kDa, (based on viscosity values) and deacetylation degree of 75%, one of them with the incorporation of non-cross-linked RES-loaded NPs and the other with the cross-linked counterpart. The final CTS concentration was set at 4% *w*/*w* and the degree of crosslinking 10%. 

Firstly, the NPs (cross-linked and non-cross-linked) were loaded with RES under the optimized conditions disclosed in the experimental design described above: RES/polymer ratio: 0.75; temperature: 39 °C, loading time: 24 h. Secondly, the hydrogel preparation was conducted similarly to the procedure recently described by us [53] that can be summarized as follows: CTS (0.4 g, 1.74 mmol of free amine groups) was charged in a round-bottom flask provided with a stirrer bar; then, an aqueous solution of tricarballylic acid (1.02 mL, 10 mg/mL, 0.05 mmol), a solution of acetic acid (0.1 mL, 52% *w*/*v*), a dispersion of resveratrol-loaded NPs (5.5 mL, RES 0.9375 mg/mL) and double-distilled water [up to a final weight of 10 g (final polymer concentration: 4% *w*/*w*)] were added in sequence. The mixture was stirred to homogenization at 40 °C during 1.5 h. The solution was cooled at 25 °C and the stirring proceeded overnight at 25 °C. 

### 3.7. Release Studies

In order to check the release of the RES-loaded NPs, two samples, cross-linked and non-cross-linked NPs, were prepared under optimum uploading conditions (RES/polymer ratio: 0.75; temperature: 39 °C, loading time: 24 h). Next, the RES released during the trials were determined by UV-Vis spectroscopy at 307 nm, similarly to the general procedure described above for the loading assays. In these cases, a mini-dialysis tube containing the NP dispersion was immersed into simulated colon fluids (pH = 6.0 ± 0.25 [62]) and the temperature was set at 37 °C. 

When the release of RES from hydrogel-NP composites was conducted, the selected RES-loaded composite was transferred to a dialysis bag (molecular weight cut-off: 8000–14,000 Da), then immersed in simulated colon fluids and gently stirred at 37 °C in a shaker incubator (Heidolph Unimax1010-Heidolph Inkubator 1000, Schwabach, Alemania). At pre-designed time intervals, aliquots were taken from the release medium and the amount of RES released was determined by UV–Vis spectroscopy at 307 nm. RES release experiments were performed in triplicate. 

## 4. Conclusions

Resveratrol (RES), a natural occurring multi-therapeutic drug, has demonstrated its capability of reducing inflammation associated with gastrointestinal tract pathologies. Our efforts have focused on the design and development of new smart micro- and nano-structured gel-like drug delivery systems as colon-specific RES depots. 

The drug was successfully encapsulated in freshly-prepared biocompatible non- and cross-linked NPs, which, in turn, were integrated into a chitosan gel matrix. The influence of two parameters, the temperature and the drug/polymer ratio, on the encapsulation efficiency (EE) in the non-cross-linked NP was compared with that found on the cross-linked systems, discovering that the temperature exerted the prevailing influence. RES-loaded CTS-NP formulations were efficiently prepared by immersing RES-loaded NPs in 4% CTS suspensions with subsequent gelation by ionic cross-linking. The study of the two drug-depots by TGA and SEM disclosed porous scaffolds with great similarities in their thermal stability patterns.

RES release from NPs was next investigated finding that non- and cross-linked NPs behaved similarly over the period of time studied (48 h). While they displayed a sustained RES release (final cumulative drug release after 48 h: 79–81%), the RES-loaded CTS-NP hydrogels, prepared at 37 °C, 4% CTS and 10% of cross-linking, showed a marked reduction in RES release rates (reductions in RES release after 48 h: 57% and 64%).

Therefore, it has been proved that, in the prepared RES depots, the chitosan network is responsible for substantially reducing the release rate of drugs, conforming such systems into a versatile tool that could potentially endow therapeutic benefits in the treatment of IBD through prolonged retention and delivery. 

## Figures and Tables

**Table 1:** Experimental encapsulation efficiency values of resveratrol loaded NPs (nanoparticles) for non-cross-linked and cross-linked systems at different RES (Resveratrol)/polymer ratios and temperatures following the experimental design ^1^.

**Experimental Parameters**		**Non Cross-Linked Loaded NPs**		
**RES/Polymer Ratio**	**Temperature (°C)**	**Sample**	**Formulation Code**	**EE (%)**
0.25	25	1	NonXr-RES/Pol_0.25_-T_25_	6.80
0.25	25	2	NonXr-RES/Pol_0.25_-T_32_	29.51
0.25	25	3	NonXr-RES/Pol_0.25_-T_39_	33.82
0.50	32	4	NonXr-RES/Pol_0.5_-T_25_	23.87
0.50	32	5	NonXr-RES/Pol_0.5_-T_32_	36.00
0.50	32	6	NonXr-RES/Pol_0.5_-T_32_	38.18
0.50	32	7	NonXr-RES/Pol_0.5_-T_39_	49.37
0.75	39	8	NonXr-RES/Pol_0.75_-T_25_	18.31
0.75	39	9	NonXr-RES/Pol_0.75_-T_32_	37.54
0.75	39	10	NonXr-RES/Pol_0.75_-T_39_	48.10
**Experimental Parameters**		**Cross-Linked Loaded NPs**		
**RES/Polymer Ratio**	**Temperature (°C)**	**Sample**	**Formulation Code**	**EE (%)**
0.25	25	11	Xr-RES/Pol_0.25_-T_25_	9.51
0.25	25	12	Xr-RES/Pol_0.25_-T_32_	43.74
0.25	25	13	Xr-RES/Pol_0.25_-T_39_	49.01
0.50	32	14	Xr-RES/Pol_0.5_-T_25_	12.57
0.50	32	15	Xr-RES/Pol_0.5_-T_32_	38.00
0.50	32	16	Xr-RES/Pol_0.5_-T_32_	40.09
0.50	32	17	Xr-RES/Pol_0.5_-T_39_	36.19
0.75	39	18	Xr-RES/Pol_0.75_-T_25_	28.46
0.75	39	19	Xr-RES/Pol_0.75_-T_32_	43.12
0.75	39	20	Xr-RES/Pol_0.75_-T_39_	44.34

^1^ Each value is the average of three samples (*p* < 0.05). EE = encapsulation efficiency; RES/polymer ratio: 0.25, 0.5 or 0.75; Temperature: 25, 32 or 39 °C.

**Table 2:** Equations yielded for the dependent variable (EE) as a function of the independent variables (RES/polymer ratio and temperature, normalized values) for the experimental design.

Equation	R^2^	Df	F	P	Std. Error
$XrEE=37.88+13.17\;T-12.34\;T^{2}+6.71\;C^{2}-5.90\;T\;C$	0.96	4.5	29.03	0.001	3.69
$NonXrEE=38.89+13.72\;T-4.07\;T^{2}+5.64\;C-7.17\;C^{2}$	0.97	4.5	49.96	0.003	2.74

C = RES/polymer ratio normalized value; C = RES/polymer ratio normalized value; XrEE = encapsulation efficiency in percentage for core cross-linked NPs; NonXrEE = encapsulation efficiency in percentage for non-cross-linked NPs

**Table 3:** Comparison of Z-average, polydispersity index (PdI), and hydrodynamic diameter (D_h_, determined by DLS) of non-cross-linked NP (Non-Xr) and stabilized NP at 20% of cross-linking (Xr) (unloaded or loaded with RES).

	Unloaded Samples [31]				Resveratrol-Loaded NPs			
Degree of Crosslinking	Sample	Z-av (± SD)	PdI (± SD)	Size (± SD) (D_h_. nm)	Sample	Z-av (± SD)	PdI (± SD)	Size (± SD) (D_h_. nm)
		(nm)				(nm)		
Non-Xr	S-01	177 (± 1)	0.14 (± 0.02)	210 (± 80)	RES-Non-Xr	115 (± 1)	0.46 (± 0.01)	170 (± 90)
Xr 20%	S-02	108 (± 1)	0.33 (± 0.01)	130 (± 70)	RES-Xr	121 (± 1)	0.27 (± 0.01)	170 (± 90)

The RES-loaded NPs were prepared at pH 7.0 according to the optimized conditions found in the present study: Sample RES-Non-Xr = Non-Xr-Res_0.75_-T_39_; RES-Xr = Xr-Res_0.75_-T_39_; Temperature = 39 °C; RES/polymer ratio = 0.75:1; loading time = 24 h.

**Table 4:** Comparison of spreadability and TGA (Thermogravimetric Analysis) data of resveratrol-loaded hydrogel-NP composites (with cross-linked NP and non-cross-linked NP.

Sample	Spreadability (diameter, cm)^a^			TGA^b^			
	t 1 min	t 30 min	Δdiameter (%)	°T_d_ (°C)	^max^T_d_ (°C)	ΔW (%)	Mass Residue at 650 °C (%)
CTS	-			110	72/297	9/58	30
Xr-CTS-RES	5	8.2	64	98	62/281	24/43	32
Non-Xr-CTS-RES	5.6	8.9	59	99	111/281	24/42	32

a: Spreadability measured in cm; ∆ diameter = change of diameter (in percentage) after 30 min; b: Onset decomposition temperature corresponding to 10% of weight loss (°Td); maximum rate decomposition temperatures (^max^T_d_) and weight loss at the respective decomposition step [ΔW(%)] determined by TGA.

## Abbreviations

TNF-α	Tumor Necrosis Factor
IL-12	Interleukin-12
IL-6	Interleukin-6
IBD	Inflammatory Bowel Disease
CD	Crohn’s Disease
UC	Ulcerative Colitis
GIT	Gastro-Intestinal Tract
DALYs	Disability-Adjusted Life Years
RES	Resveratrol
ADME-T	Absorption, Distribution, Metabolism, Excretion and Toxicity
DDS	Drug Delivery Systems
NPs	Nanoparticles
MNP	Micro-Nanoparticles
ADME-T	Absorption, Distribution, Metabolism, Excretion and Toxicity
pHEMA	poly(2-hydroxyethyl methacrylate)
pDMAEMA	poly(*N,N*-dimethylaminoethyl methacrylate)
EE	Encapsulation Efficiency
LCST	Lower Critical Solution Temperature
CTS	Chitosan
DCNa	Diclofenac Sodium
CEBAS-CSIC	Segura’s Center of Edaphology and Applied Biology
UV-Vis	Ultraviolet Visible
SEM	Scanning Electron Microscopy
CITIUS	General Research Services of the University of Seville
D_h_	Average Diameter
PdI	Polydispersity Index
DLS	Dynamic Light Scattering
TGA	Thermogravimetric Analysis
DTG	Derivative Thermogravimetric Analysis
DMDOO	1,8-dimaleimide-3,6-dioxaoctane
DMSO	Dimethyl sulfoxide
